# Predictive role of preoperative parameters in LAMP outcomes for myelopathy caused by COPLL

**DOI:** 10.1186/s12891-025-08577-0

**Published:** 2025-04-08

**Authors:** Hao Yuan, Wei Lei, Wenping Li, Yunlong Zhou, Xufeng Jia, Daxiong Feng, Fei Lei

**Affiliations:** 1 Spinal Surgery Department, The Affiliated Hospital of Southwest Medical University, Luzhou, Sichuan PR China; 2 Department of orthopedics, The Affiliated Hospital of Southwest Medical University, Luzhou, China; 3 Orthopedics Department, Luzhou Maternal & Child Health Hospital, Luzhou, China; 4 Orthopedics Department, The People’s Hospital of Leshan, Leshan, China; 5 Orthopedics Department, The People’s Hospital of Jianyang City, Jianyang, China

**Keywords:** Predictive role, JOACMEQ, K-line, Torg-Pavlov ratio, LAMP, COPLL

## Abstract

**Objectives:**

This study aims to assess the predictive value of preoperative parameters on recovery outcomes in patients with myelopathy caused by cervical ossification of the posterior longitudinal ligament (COPLL) undergoing laminoplasty (LAMP).

**Methods:**

A retrospective analysis was performed on myelopathy patients caused by COPLL who underwent LAMP between 2017 and 2020. Preoperative variables, including basic epidemiological characteristics, comorbidities, functional scores, K-line-related parameters, Torg-Pavlov ratio, maximal SCOR and COPLL shape, were analyzed for their predictive influence on postoperative outcomes in cervical spine function, upper and lower extremity function, bladder function, and quality of life (QOL). Binary logistic regression model analyses were used to evaluate predictive accuracy.

**Results:**

A total of 84 patients were included in the study. Preoperative parameters were significant predictors of postoperative improvement following LAMP surgery for myelopathy caused by COPLL. K-line-related factors, including K-line (-) (AUC = 0.80) and K-line on sagittal T1WI (-) (AUC = 0.76), were important predictors of cervical spine function improvement. Preoperative QOL scores (AUC = 0.78) also played a significant role in predicting cervical spine function improvement. For upper extremity function, preoperative upper extremity scores were a key predictor (AUC = 0.79), while C4-C6 K-line (-) (AUC = 0.81) was also a relevant factor. Similarly, preoperative lower extremity scores were crucial for predicting lower extremity function improvement (AUC = 0.85), and preoperative QOL scores were significant predictors of QOL improvement (AUC = 0.78). Other parameters, such as the Torg-Pavlov ratio, maximal SCOR, and the shape of COPLL, provided supplementary predictive value, though their influence was secondary to JOACMEQ scores and K-line parameters. Bladder function showed minimal postoperative improvement, with preoperative bladder status and the Torg-Pavlov ratio at C5 being the primary predictors for bladder improvement. Overall, preoperative K-line findings, JOACMEQ scores, and spinal canal measurements provided valuable guidance for postoperative expectations and surgical planning.

**Conclusions:**

Preoperative K-line parameters and JOACMEQ scores are robust predictors of functional recovery in myelopathy patients caused by COPLL undergoing LAMP. While Torg-Pavlov ratio, maximal SCOR, and the shape of COPLL offer additional predictive value for overall recovery, they remain useful for preoperative surgical planning. These findings emphasize the importance of comprehensive preoperative assessment to optimize outcomes.

## Introduction

Cervical ossification of the posterior longitudinal ligament (COPLL) is a pathological condition characterized by the ossification of the posterior longitudinal ligament in the cervical spine, leading to spinal cord compression and subsequent myelopathy. Myelopathy is the primary clinical manifestation of COPLL, presenting with symptoms such as limb numbness, weakness, gait instability, and impaired fine motor skills. Radiculopathy may also occur in some cases. Due to the progressive nature of myelopathy, timely surgical intervention is crucial to alleviate spinal cord compression, restore neurological function, and improve patients’ quality of life (QOL). Laminoplasty (LAMP) is a widely used surgical approach for COPLL, effectively relieving spinal cord compression by reshaping the lamina to create additional space within the spinal canal, making it a preferred option for treating COPLL-associated myelopathy [1].

Predicting the effectiveness of LAMP for COPLL requires consideration of various preoperative factors, with the K-line being the most renowned predictive tool. The K-line, drawn on lateral cervical X-rays between the midpoints of the spinal canal at the C2 and C7 levels, classifies patients into K-line positive (K(+)) and K-line negative (K(-)), with studies showing that K(-) status is unfavorable for neurological recovery in COPLL patients after LAMP [2].

To enhance the predictive accuracy of the K-line, researchers have proposed various modifications. For example, reducing the segmental range of the K-line to C4-C6^3^, altering the patient’s position during K-line measurement [3], and utilizing MRI to better clarify spinal cord compression [4]. One study reviewed 113 K(+) COPLL patients and grouped them based on the K-line in the flexion position, finding that the Japanese Orthopaedic Association (JOA) recovery rates in the modified K(-) group were significantly lower than those in the modified K(+) group, indicating that the modified K-line is an important predictor of functional recovery after laminectomy [3]. Additionally, research has focused on the impact of the K-line tilt angle on LAMP outcomes. For example, a study by Kenichiro Sakai et al. on 62 K-line positive (K(+)) COPLL patients found that a K-line tilt angle greater than 20° is a preoperative risk factor for kyphotic deformity after LAMP [5].

Besides the K-line, the spinal canal occupying ratio (SCOR) and Pavlov ratio have also been shown to be key predictors of the effectiveness of LAMP. SCOR, the ratio of the maximum ossification thickness to the anteroposterior diameter of the spinal canal, is accurately measured using axial CT imaging. A higher SCOR (> 50–60%) usually indicates the need for more aggressive anterior decompression surgery rather than LAMP [6]. Studies have shown that an average Pavlov ratio of less than 0.65 is a high-risk factor for postoperative upper limb palsy following LAMP [7].

Despite numerous studies exploring the predictive value of different preoperative indicators for the effectiveness of LAMP, several gaps remain. First, few studies comprehensively examine which factors—such as the degree of COPLL invasion, the extent of spinal canal stenosis, and the K-line and its modifications—are the best predictors of LAMP outcomes [8]. Second, most studies use the JOA or mJOA score to assess neurological function recovery, but few design studies to evaluate multiple aspects of recovery such as cervical function and quality of life [9].

This study aims to address these gaps by analyzing a cohort of patients with myelopathy caused by COPLL undergoing LAMP and comparing preoperative variables, including the K-line and its modifications, SCOR, Pavlov ratio, and other demographic and clinical factors. To the best of our knowledge, this study is the first to directly compare the predictive value of K-line and its modified parameters for surgical outcomes in COPLL patients undergoing LAMP. This may help optimize surgical planning and improve postoperative recovery and quality of life for COPLL patients.

## Methods

### Patient selection

This study was approved by the Clinical Trial Ethics Committee of the Affiliated Hospital of Southwest Medical University (registration number: KY2023361) on August 4, 2023. Patients with COPLL who underwent LAMP and completed prospective the Japanese Orthopaedic Association Cervical Myelopathy Evaluation Questionnaire (JOACMEQ) assessments at four medical centers between January 1, 2017, and December 31, 2020, were included in this study. The JOACEMQ scores were reassessed between January to June 2024.

Inclusion criteria were: (1) clinical manifestations of myelopathy, such as limb numbness or weakness, unsteady gait, and clumsiness; (2) preoperative imaging evidence of COPLL in the cervical spine with spinal cord compression; (3) undergoing LAMP decompression with arch plate fixation between January 1, 2017, and December 31, 2020; and (4) fully completed preoperative JOACEMQ assessments. Exclusion criteria included: (1) Comorbidities that significantly impact JOACMEQ scores, including cardiovascular disease, respiratory disorders, endocrine diseases, rheumatoid arthritis, psychiatric disorders, severe urinary tract conditions, uremia, and others; (2) cervical trauma or tumors; (3) a history of cervical surgery; (4) patients who underwent concomitant thoracic or lumbar spine surgery; and (5) incomplete postoperative questionnaires.

Surgeries were performed by multiple experienced senior spine surgeons across four medical centers, each with a minimum of 15 years of experience in spinal surgeries and extensive expertise in performing laminoplasty procedures.

### Baseline demographic and clinical data

**Fig. 1:**
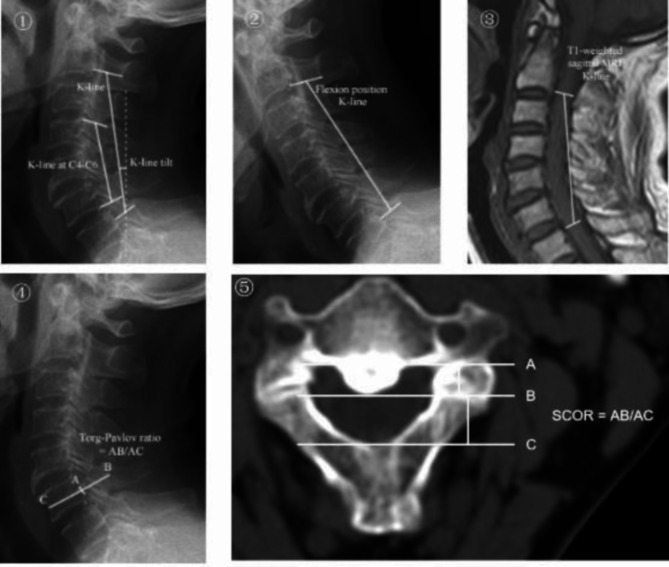
①. K-line is a straight line connecting the midpoints of the spinal canal at C2 and C7 on a neutral lateral cervical X-ray. K-line tilt refers to the angle between the K-line and a vertical line. K-line at C4-6 is a straight line connecting the midpoints of the spinal canal from C4 to C6. ②. K-line in the neck-flexed position is a straight line connecting the midpoints of the spinal canal at C2 and C7 on a flexion lateral cervical X-ray. ③. K-line on sagittal T1WI is defined as a line connecting the midpoints of the spinal cord at C2 and C7 on T1-weighted sagittal MRI. ④. Torg-Pavlov ratio is the ratio of the sagittal diameter of the spinal canal (AB) to the sagittal diameter of the vertebral body (AC). ⑤. SCOR is the ratio of the maximum thickness of COPLL (AB) to the anteroposterior diameter of the spinal canal (AC) at the same level

Demographic data, including age, gender, Body Mass Index (BMI), among others, were recorded for patients meeting the inclusion criteria. Two senior spine surgeons independently measured the K-line, K-line tilt, C4-C6 K-line, K-line in the neck-flexed position, K-line on sagittal T1-weighted imaging (T1WI), Torg–Pavlov ratio from C2 to C7, and the maximal SCOR for each subject. The measurement method is illustrated in Fig. 1. Additionally, the sagittal shape of the ossified lesion on X-rays was classified as either plateau-shaped or hill-shaped and documented [10]. If the two surgeons disagreed on a measurement, a third senior spine surgeon conducted additional measurements, and a consensus was reached through discussion. If the minimum distance between the K-line on sagittal T1WI and the anterior compression factor is ≤ 4 mm, the K-line on sagittal T1WI is considered positive. The criteria for determining positive results for other modified K-lines were consistent with those of the original K-line.

The JOACMEQ was used pre- and postoperatively to assess cervical spine function, upper extremity function, lower extremity function, bladder function, and patient QOL [11]. Based on the User’s Guide for the JOACMEQ [12], as well as studies by Chien et al. [13] and Takeshita et al. [14], surgery was considered effective if it resulted in an improvement in JOACMEQ scores of at least 2.5 points in cervical function, 13.0 points in upper extremity function, 9.35 points in lower extremity function, 6.0 points in bladder function, 9.5 points in QOL, or if the final follow-up score exceeded 90 points in any domain.

### Grouping and statistical analysis

patients were stratified into improvement and non-improvement groups based on surgical outcomes for cervical spine function, upper extremity function, lower extremity function, bladder function, and QOL respectively. Baseline demographic characteristics were compared between the improvement and non-improvement groups to assess comparability. Clinical variables, including the K-line, C4-C6 K-line, K-line in the neck-flexed position, K-line on sagittal T1WI, Torg–Pavlov ratio at C2-C7, minimum Torg–Pavlov ratio, average Torg–Pavlov ratio, maximum SCOR, and COPLL shape, were also compared between groups to identify significant differences. Categorical variables were analyzed using chi-square tests, while independent t-tests were employed for continuous variables.

All continuous variables were standardized by subtracting the mean and dividing by the standard deviation. Categorical variables were encoded as follows: positive (modified) K-line was encoded as 0 and negative (modified) K-line as 1; plateau-shaped COPLL as 0 and hill-shaped COPLL as 1; female as 0 and male as 1; the presence of any comorbidities or lifestyle factors was encoded as 1, otherwise as 0. Surgical outcome (improvement or non-improvement) served as the dependent variable in our analysis. Binary logistic regression was then performed on variables with significant inter-group differences to assess their predictive accuracy (area under the curve (AUC)) and effect size (standardized odds ratio (OR) and regression coefficient) on surgical outcomes. A *P*-value of < 0.05 was considered statistically significant for all tests.

## Result

### Baseline and functional scores

Table 1 presents baseline characteristics and functional scores, including both preoperative and postoperative assessments. A total of 84 patients were included in the final cohort. The cohort’s mean age was 59.79 ± 8.55 years, with 48.81% male patients. Symptom duration ranged with a mean of 23.65 ± 18.74 months. Diabetes and hypertension were present in 17.86% and 28.57% of patients, respectively.

**Table 1:** Baseline characteristics of the cohort

Variables	Mean ± SD or number (percentage)	Range (min - max)
Age (years)	59.79 ± 8.55	42.47–80.72
Male	41(48.81%)	N/A
BMI (kg/cm²)	23.62 ± 2.36	19.14–28.93
Duration (months)	23.65 ± 18.74	3.00–78.00
Diabetes	15.00(17.86%)	N/A
Hypertension	24.00(28.57%)	N/A
Smoker	14.00(16.67%)	N/A
Drinker	12.00(14.29%)	N/A
Prostatic hyperplasia	13.00(15.48%)	N/A
Thoracic OYL Association	26.00(30.95%)	N/A
Follow-up time (years)	5.73 ± 0.88	4.48–7.43
Preoperative cervical spine function	63.04 ± 24.73	0.00–100.00
Postoperative cervical spine function	85.48 ± 19.48	45.00–100.00
Preoperative upper extremity function	64.91 ± 21.94	0.00–100.00
Postoperative upper extremity function	74.31 ± 12.02	52.63–100.00
Preoperative lower extremity function	55.47 ± 21.87	0.00–100.00
Postoperative lower extremity function	87.82 ± 16.14	40.91–100.00
Preoperative bladder function	93.08 ± 12.16	37.5–100.00
Postoperative bladder function	95.83 ± 9.27	37.5–100.00
Preoperative QOL	39.17 ± 18.81	5.21–71.88
Postoperative QOL	59.31 ± 15.23	22.92–83.33
C2-C7 Cobb angel (°)	10.44 ± 8.54	-5.30–32.60
Torg–Pavlov ratio C3	0.82 ± 0.16	0.46–1.43
Torg–Pavlov ratio C4	0.79 ± 0.13	0.54–1.17
Torg–Pavlov ratio C5	0.77 ± 0.14	0.54–1.16
Torg–Pavlov ratio C6	0.82 ± 0.14	0.55–1.15
Torg–Pavlov ratio C7	0.86 ± 0.16	0.61–1.23
Minimal Torg–Pavlov ratio	0.71 ± 0.12	0.46–1.06
Average Torg–Pavlov ratio	0.81 ± 0.12	0.63–1.16
K-line (-)	25.00 (29.76%)	N/A
K-line tilt (°)	9.96 ± 6.87	1.20–28.60
C4-C6 K-line (-)	29.00 (34.52%)	N/A
K-line in the neck-flexed position (-)	35.00 (41.67%)	N/A
K-line on sagittal T1WI (-)	30.00 (35.71%)	N/A
Maximal SCOR(%)	44.75 ± 0.11	0.22–0.69
Shape of COPLL (hill-shaped)	20.00(23.81%)	N/A
C5 palsy	9.00(10.71%)	N/A
Cerebrospinal fluid leak	3.00(3.57%)	N/A
Surgical site infections	4.00(4.76%)	N/A
Axial pain	16.00(19.05%)	N/A

BMI body mass index; OYL ossification of the yellow ligament; SCOR spinal canal occupying ratio; COPLL cervical ossification of the posterior longitudinal ligament; QOL quality of life; T1WI T1-weighted imaging; SD standard deviation. Data are presented as mean ± standard deviation unless stated to be number and percentage, n (%). * indicates a statistically significant difference compared with the preoperative functional score

Postoperative complications were monitored in this cohort, with the most common being axial pain, which occurred in 19.05% (16 patients) of the cohort. Other complications included C5 palsy in 10.71% (9 patients), cerebrospinal fluid leaks in 3.57% (3 patients), and surgical site infections in 4.76% (4 patients).

Postoperative scores for cervical spine, upper extremity, lower extremity, and QOL showed significant improvements compared to preoperative values (*p* < 0.05). In contrast, bladder function scores did not exhibit a statistically significant change following surgery, suggesting a limited response to LAMP surgery in this area. These findings highlight the variable impact of the surgery on different functional domains, with substantial recovery observed in all areas except bladder function.

### Comparison of preoperative parameters by outcome groups

Table 2 compares preoperative variables between patients with and without postoperative improvement in the domains of cervical spine function, upper extremity function, lower extremity function, bladder function, and QOL. In the domains of cervical spine function, upper extremity function, lower extremity function, and QOL, the improvement group consistently exhibited lower K-line tilt angles, reduced negativity rates for various K-lines, and better preoperative function scores. Additionally, for upper extremity function, the improvement group exhibited wider Pavlov ratios and a lower prevalence of hill-shaped COPLL. And in Lower extremity function improvement group exhibited a reduced maximal SCOR, along with a decreased prevalence of both hill-shaped COPLL and thoracic OYL association. With respect to QOL, patients in the improvement group were also younger. Regarding bladder function, significant differences were observed only in preoperative bladder function scores and the Torg-Pavlov ratio at C5, with higher values noted in the improvement group.

**Table 2:** Comparison of preoperative parameters by outcome groups

	Cervical spine function			Upper extremity function			Lower extremity function			Bladder function			Quality of life	
	Improvement (*n* = 61)	Non-improvement (*n* = 23)		Improvement (*n* = 53)	Non-improvement (*n* = 31)		Improvement (*n* = 61)	Non-improvement (*n* = 23)		Improvement (*n* = 80)	Non-improvement (*n* = 4)		Improvement (*n* = 57)	Non-improvement (*n* = 27)
Age (years)	58.89 ± 8.69	62.18 ± 8.83		58.36 ± 8.78	62.23 ± 7.84		58.73 ± 8.20	62.60 ± 9.18		59.81 ± 8.59	59.39 ± 10.27		58.40 ± 8.01	62.72 ± 9.20^*^
Male	31 (50.82%)	10 (43.48%)		25 (47.17%)	15 (48.39%)		30 (49.18%)	10 (47.83%)		40 (50.00%)	1 (25.00%)		28 (49.12%)	13 (48.15%)
BMI (kg/cm²)	23.41 ± 2.58	24.17 ± 2.56		23.55 ± 2.05	23.73 ± 2.88		23.49 ± 2.51	23.95 ± 1.98		23.63 ± 2.39	23.47 ± 2.51		23.49 ± 2.32	23.88 ± 2.51
Duration (months)	22.84 ± 18.06	25.80 ± 18.41		23.68 ± 19.14	23.61 ± 18.33		23.36 ± 19.59	24.43 ± 16.65		23.15 ± 18.24	33.75 ± 28.43		22.30 ± 19.38	26.52 ± 17.29
Diabetes	12 (19.67%)	3 (13.04%)		9 (16.98%)	6 (19.35%)		9 (14.75%)	6 (26.09%)		14 (17.50%)	1 (25.00%)		10 (17.54%)	5 (18.52%)
Hypertension	16 (26.23%)	8 (34.78%)		12 (22.64%)	12 (38.71%)		16 (26.23%)	8 (34.78%)		23 (28.75%)	1 (25.00%)		14 (24.56%)	10 (37.04%)
Smoker	9 (14.75%)	5 (21.74%)		6 (11.32%)	8 (25.81%)		9 (14.75%)	5 (21.74%)		14 (17.50%)	0 (0.00%)		7 (12.28%)	7 (25.93%)
Drinker	10 (16.39%)	2 (8.70%)		5 (9.43%)	7 (22.58%)		7 (11.48%)	5 (21.74%)		12 (15.00%)	0 (0.00%)		8 (14.04%)	4 (14.81%)
Prostatic hyperplasia	12 (19.67%)	5 (21.74%)		11 (20.75%)	6 (19.35%)		13 (21.31%)	4 (17.39%)		16 (20.0%)	1 (25.0%)		11 (19.3%)	6 (22.22%)
Thoracic OYL Association	19 (31.15%)	7 (30.43%)		16 (30.19%)	10 (32.26%)		13 (21.31%)	13 (56.52%)^*^		26 (32.50%)	0 (0.00%)		17 (29.82%)	9 (33.33%)
Preoperative cervical spine function	63.26 ± 21.14	62.46 ± 25.96		69.26 ± 18.79	52.41 ± 26.30^*^		65.08 ± 27.03	57.98 ± 23.71		63.18 ± 23.71	60.24 ± 27.03		64.44 ± 17.06	60.08 ± 27.74
Preoperative upper extremity function	67.60 ± 10.36	57.78 ± 24.02^*^		71.27 ± 18.67	54.04 ± 23.59^*^		68.52 ± 22.02	55.34 ± 20.24^*^		65.12 ± 22.02	60.71 ± 20.24		71.49 ± 10.97	51.02 ± 24.61^*^
Preoperative lower extremity function	58.07 ± 11.70	48.57 ± 14.39^*^		62.69 ± 13.48	43.13 ± 23.66^*^		63.07 ± 20.36	35.31 ± 22.26^*^		55.63 ± 21.92	52.27 ± 23.60		60.96 ± 12.53	43.88 ± 24.42^*^
Preoperative bladder function	94.69 ± 15.79	88.81 ± 12.98		95.77 ± 15.56	88.48 ± 14.32		94.55 ± 3.64	89.18 ± 13.75^*^		93.22 ± 11.22	90.28 ± 14.79^*^		96.06 ± 7.59	86.79 ± 13.64
Preoperative QOL	41.37 ± 11.47	33.33 ± 19.66^*^		46.10 ± 10.97	27.32 ± 19.56^*^		43.46 ± 14.37	27.79 ± 19.43^*^		39.15 ± 18.53	39.57 ± 17.76		42.77 ± 5.13	31.57 ± 20.41^*^
Torg–Pavlov ratio C3	0.83 ± 0.16	0.78 ± 0.14		0.86 ± 0.15	0.75 ± 0.16^*^		0.83 ± 0.15	0.79 ± 0.17		0.82 ± 0.12	0.82 ± 0.16		0.83 ± 0.17	0.79 ± 0.12
Torg–Pavlov ratio C4	0.82 ± 0.12	0.72 ± 0.14		0.83 ± 0.12	0.72 ± 0.13^*^		0.80 ± 0.13	0.76 ± 0.14		0.80 ± 0.09	0.69 ± 0.13		0.80 ± 0.13	0.76 ± 0.13
Torg–Pavlov ratio C5	0.80 ± 0.13	0.69 ± 0.14		0.81 ± 0.12	0.70 ± 0.15^*^		0.78 ± 0.13	0.74 ± 0.16		0.78 ± 0.10	0.69 ± 0.14^*^		0.78 ± 0.14	0.74 ± 0.15
Torg–Pavlov ratio C6	0.83 ± 0.13	0.79 ± 0.18		0.86 ± 0.11	0.75 ± 0.16^*^		0.83 ± 0.14	0.79 ± 0.16		0.83 ± 0.03	0.78 ± 0.16		0.82 ± 0.13	0.81 ± 0.18
Torg–Pavlov ratio C7	0.87 ± 0.14	0.83 ± 0.19		0.89 ± 0.14	0.81 ± 0.15^*^		0.87 ± 0.15	0.83 ± 0.17		0.87 ± 0.03	0.72 ± 0.13		0.86 ± 0.12	0.84 ± 0.21
minimal Torg–Pavlov ratio	0.74 ± 0.12	0.63 ± 0.13		0.76 ± 0.10	0.63 ± 0.13^*^		0.72 ± 0.12	0.68 ± 0.14		0.71 ± 0.13	0.64 ± 0.03		0.73 ± 0.13	0.69 ± 0.12
average Torg–Pavlov ratio	0.83 ± 0.11	0.76 ± 0.14		0.85 ± 0.09	0.74 ± 0.13^*^		0.82 ± 0.11	0.78 ± 0.14		0.81 ± 0.05	0.75 ± 0.12		0.82 ± 0.11	0.79 ± 0.14
C2-C7 Cobb angel (°)	10.72 ± 7.63	9.80 ± 10.44		11.02 ± 10.74	9.44 ± 7.11		11.04 ± 10.59	8.85 ± 7.72		10.51 ± 8.73	9.04 ± 5.52		10.95 ± 10.08	9.36 ± 7.86
K-line (-)	10 (16.39%)	15 (65.22%) ^*^		4 (7.55%)	21 (67.74%)^*^		13 (21.31%)	12 (52.17%)^*^		25 (31.25%)	0 (0.00%)		10 (17.54%)	15 (55.56%)^*^
K-line tilt (°)	7.84 ± 4.21	15.58 ± 9.12^*^		8.19 ± 4.26	12.99 ± 9.18*		9.08 ± 5.49	12.30 ± 9.38^*^		9.69 ± 7.58	15.36 ± 6.88		7.56 ± 4.05	15.02 ± 8.73^*^
C4-C6 K-line (-)	14 (22.95%)	15 (65.22%) ^*^		6 (11.32%)	23 (74.19%)^*^		15 (24.59%)	14 (60.87%)^*^		29 (36.25%)	0 (0.00%)		12 (21.05%)	17 (62.96%)^*^
K-line in the neck-flexed position (-)	20 (32.79%)	15 (65.22%) ^*^		12 (22.64%)	23 (74.19%)^*^		19 (31.15%)	16 (69.57%)^*^		35 (43.75%)	0 (0.00%)		18 (31.58%)	17 (62.96%)^*^
K-line on sagittal T1WI (-)	15 (24.59%)	15 (65.22%) ^*^		7 (13.21%)	23 (74.19%)^*^		16 (26.23%)	14 (60.87%)^*^		30 (37.50%)	0 (0.00%)		11 (19.30%)	19 (70.37%)^*^
Maximal SCOR (%)	45.98 ± 10.41	41.19 ± 12.35		43.11 ± 9.29	44.13 ± 13.59		46.22 ± 13.92	40.85 ± 11.24^*^		44.28 ± 7.89	54.53 ± 9.50		45.53 ± 9.50	43.10 ± 13.92
COPLL shape (hill-shaped)	13 (21.31%)	7 (30.43%)		5 (9.43%)	15 (48.39%)^*^		9 (14.75%)	11 (47.83%)^*^		20 (25.00%)	0 (0.00%)		13 (22.81%)	7 (25.93%)

BMI body mass index; OYL ossification of the yellow ligament; SCOR spinal Canal occupying ratio; COPLL cervical ossification of the posterior longitudinal ligament; QOL quality of life; T1WI T1-weighted imaging. * Indicates a statistically significant difference in the non-improvement group as compared to the improvement group within the same domain. Data are presented as mean ± standard deviation unless stated to be number and percentage, N (%)

### Comparison of preoperative parameters’ influence on recovery across functional scores

#### Cervical spine function

**Fig. 2:**
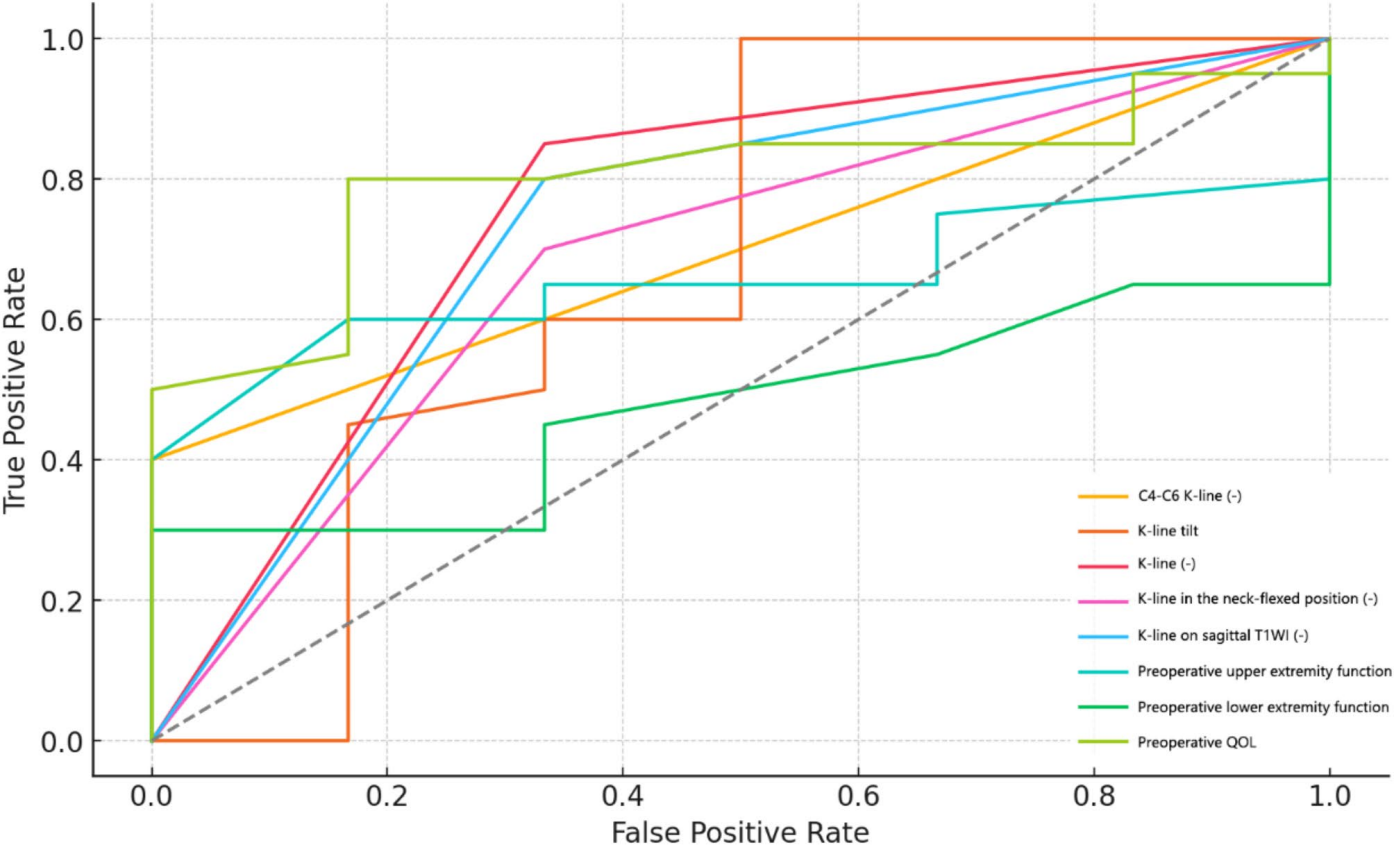
ROC curves for cervical spine function recovery after LAMP surgery. QOL quality of life; T1WI T1-weighted imaging

Table 3 summarizes the key predictors for cervical spine function recovery. Among them, K-line was identified as the strongest predictor, with an AUC of 0.80, a negative coefficient (-2.07), and an odds ratio (OR) of 0.13, suggesting that positive K-line findings are associated with better postoperative outcomes. Other K-line-related parameters showed similar trends but with lower predictive values, including K-line on sagittal T1WI, C4-C6 K-line, and K-line in the neck-flexed position. Additionally, K-line tilt demonstrated predictive significance (AUC = 0.70, coefficient = -1.26, OR = 0.26), indicating that smaller tilt angles correlate with improved recovery. Preoperative QOL also proved to be a significant predictor (AUC = 0.78, coefficient = 1.12), with higher baseline QOL scores linked to better outcomes. Figure 2 further illustrates the predictive strength of these parameters.

**Table 3:** Binary logistic regression for factors influencing cervical spine function after LAMP surgery

Variable	Odds Ratio	Coefficient	AUC
K-line (-)	0.13	-2.07	0.80
Preoperative QOL	3.07	1.12	0.78
K-line on sagittal T1WI (-)	0.14	-1.94	0.76
C4-C6 K-line (-)	0.15	-1.90	0.74
K-line in the neck-flexed position (-)	0.28	-1.87	0.70
K-line tilt	0.26	-1.26	0.70
Preoperative upper extremity function	2.27	0.82	0.66
Preoperative lower extremity function	1.32	0.28	0.48

QOL quality of life; T1WI T1-weighted imaging; AUC area under the curve

#### Upper extremity function

**Fig. 3:**
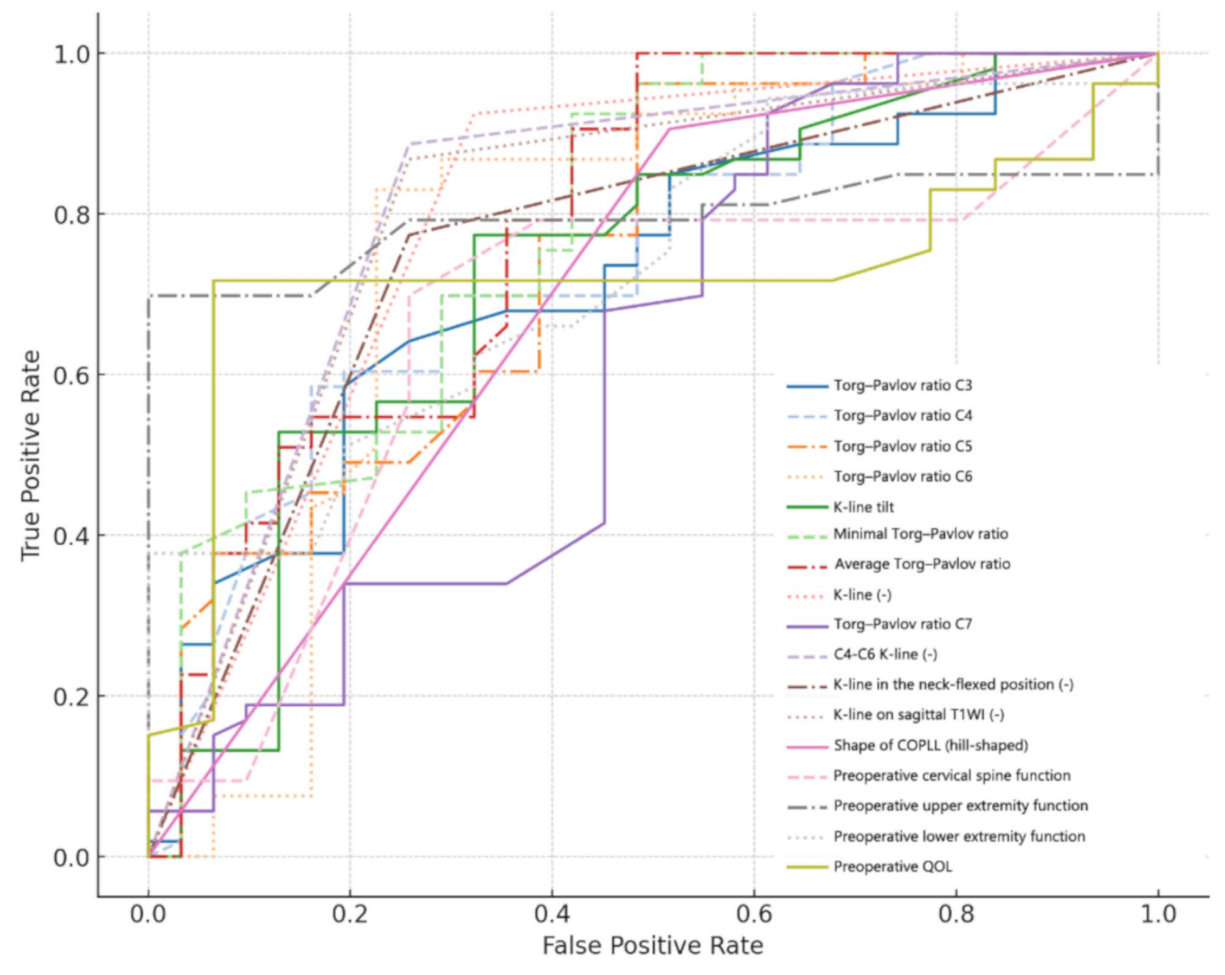
ROC curves for upper extremity function recovery after LAMP surgery. COPLL cervical ossification of the posterior longitudinal ligament; QOL quality of life; T1WI T1-weighted imaging

As shown in Table 4; Fig. 3, K-line-related parameters, including C4-C6 K-line, K-line on sagittal T1WI, general K-line and K-line in the neck-flexed position demonstrated strong predictive associations with upper extremity recovery. Each of these parameters exhibited negative coefficients and high AUC values. The K-line tilt showed moderate predictive ability (AUC = 0.73, OR = 0.50). Among functional scores, preoperative upper extremity function was the strongest predictor (AUC = 0.79, coefficient = 1.21, OR = 3.36), suggesting that higher baseline scores are associated with better recovery potential. Preoperative lower extremity function also contributed moderately to prediction. Furthermore, Pavlov ratios (AUCs ranging from 0.78 to 0.62) were significant, with ORs greater than 2.0, indicating that wider spinal canals are associated with improved recovery. Additionally, the presence of a hill-shaped COPLL also predicted reduced postoperative upper extremity function improvement (coefficient − 1.66, AUC 0.70).

**Table 4:** Binary logistic regression for factors influencing upper extremity function after LAMP surgery

Variable	Odds Ratio	Coefficient	AUC
C4-C6 K-line (-)	0.25	-1.37	0.81
K-line on sagittal T1WI (-)	0.10	-2.26	0.80
K-line (-)	0.09	-2.39	0.80
Preoperative upper extremity function	3.36	1.21	0.79
Minimal Torg–Pavlov ratio	3.08	1.12	0.78
Average Torg–Pavlov ratio	2.87	1.05	0.78
Torg–Pavlov ratio C6	2.44	0.89	0.76
K-line in the neck-flexed position (-)	0.16	-1.81	0.76
Torg–Pavlov ratio C5	2.36	0.86	0.75
Torg–Pavlov ratio C4	2.52	0.92	0.74
Preoperative lower extremity function	2.38	0.87	0.73
K-line tilt	0.50	-0.69	0.73
Torg–Pavlov ratio C3	2.20	0.79	0.72
Preoperative QOL	2.72	1.00	0.72
Shape of COPLL (hill-shaped)	0.19	-1.66	0.70
Preoperative cervical spine function	1.86	0.62	0.66
Torg–Pavlov ratio C7	2.17	0.78	0.62

COPLL cervical ossification of the posterior longitudinal ligament; QOL quality of life; T1WI T1-weighted imaging; AUC area under the curve

#### Lower extremity function

**Fig. 4:**
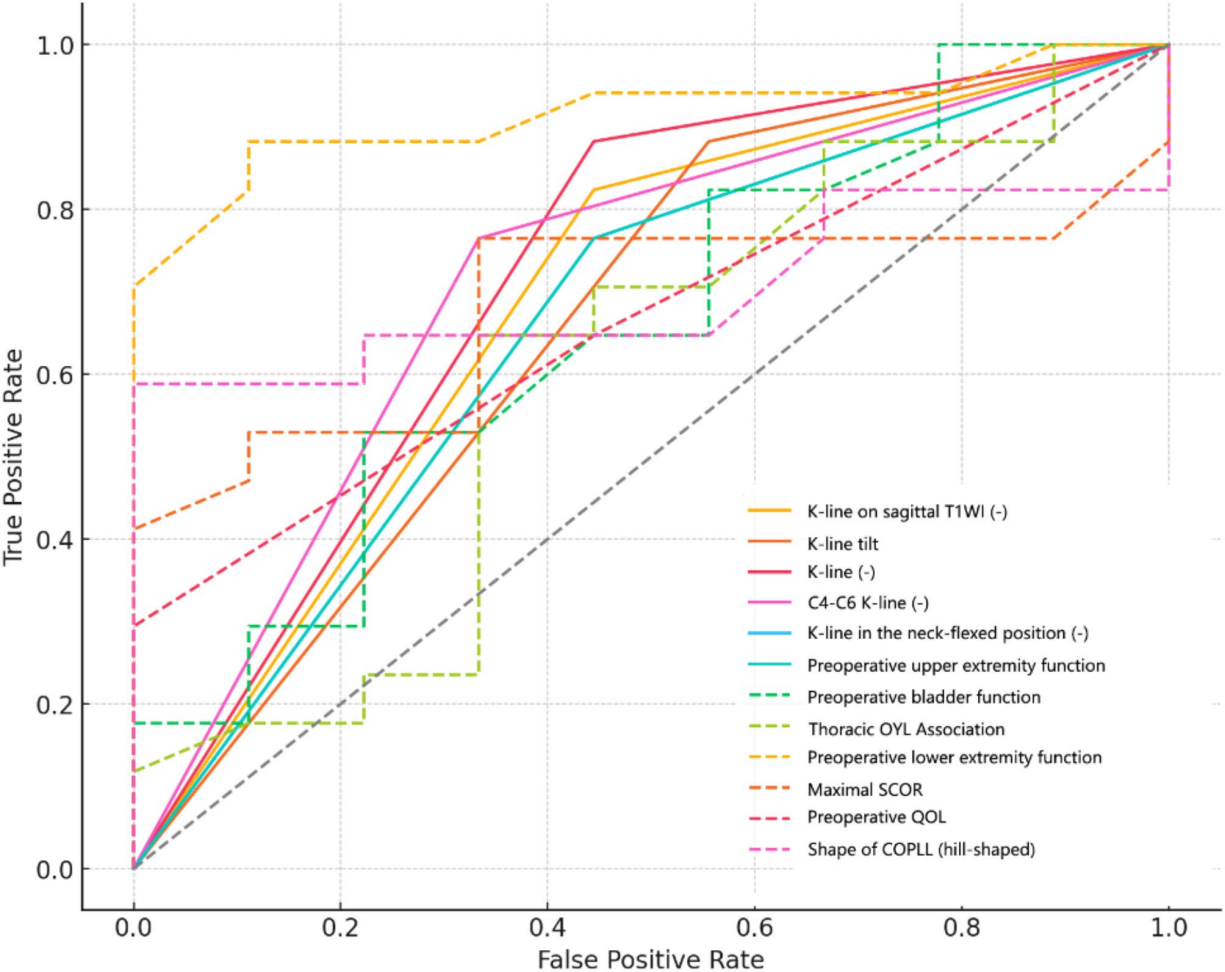
ROC curves for lower extremity function recovery after LAMP surgery. SCOR spinal canal occupying ratio; COPLL cervical ossification of the posterior longitudinal ligament; QOL quality of life; T1WI T1-weighted imaging

Table 5; Fig. 4 summarize factors predicting lower extremity function recovery. The preoperative lower extremity function score was the strongest predictor (AUC = 0.85, coefficient = 1.15), demonstrating that higher scores correlate with improved outcomes. General K-line, K-line on sagittal T1WI, C4-C6 K-line, K-line in the neck-flexed position, K-line tilt showed predictive relevance (AUC = 0.78, 0.72, 0.71, 0.71, 0.70; coefficients = -0.87, -1.22, -1.15, -1.20, -0.45), with negative K-line values linked to poorer recovery. Hill-shaped COPLL (AUC = 0.72, OR = 0.35) and Maximal SCOR (AUC = 0.70, OR = 0.46) provided additional moderate predictive contributions.

**Table 5:** Binary logistic regression for factors influencing lower extremity function after LAMP surgery

Variable	Odds Ratio	Coefficient	AUC
Preoperative lower extremity function	3.16	1.15	0.85
K-line (-)	0.42	-0.87	0.78
K-line on sagittal T1WI (-)	0.29	-1.22	0.72
Shape of COPLL (hill-shaped)	0.35	-1.04	0.72
C4-C6 K-line (-)	0.32	-1.15	0.71
K-line in the neck-flexed position (-)	0.30	-1.20	0.71
Maximal SCOR	0.46	-0.77	0.70
K-line tilt	0.64	-0.45	0.70
Preoperative upper extremity function	1.94	0.66	0.68
Preoperative QOL	1.69	0.52	0.61
Preoperative bladder function	2.16	0.77	0.64
Thoracic OYL Association	0.28	-1.28	0.63

SCOR spinal canal occupying ratio; COPLL cervical ossification of the posterior longitudinal ligament; QOL quality of life; T1WI T1-weighted imaging; AUC area under the curve

#### Bladder function

**Fig. 5:**
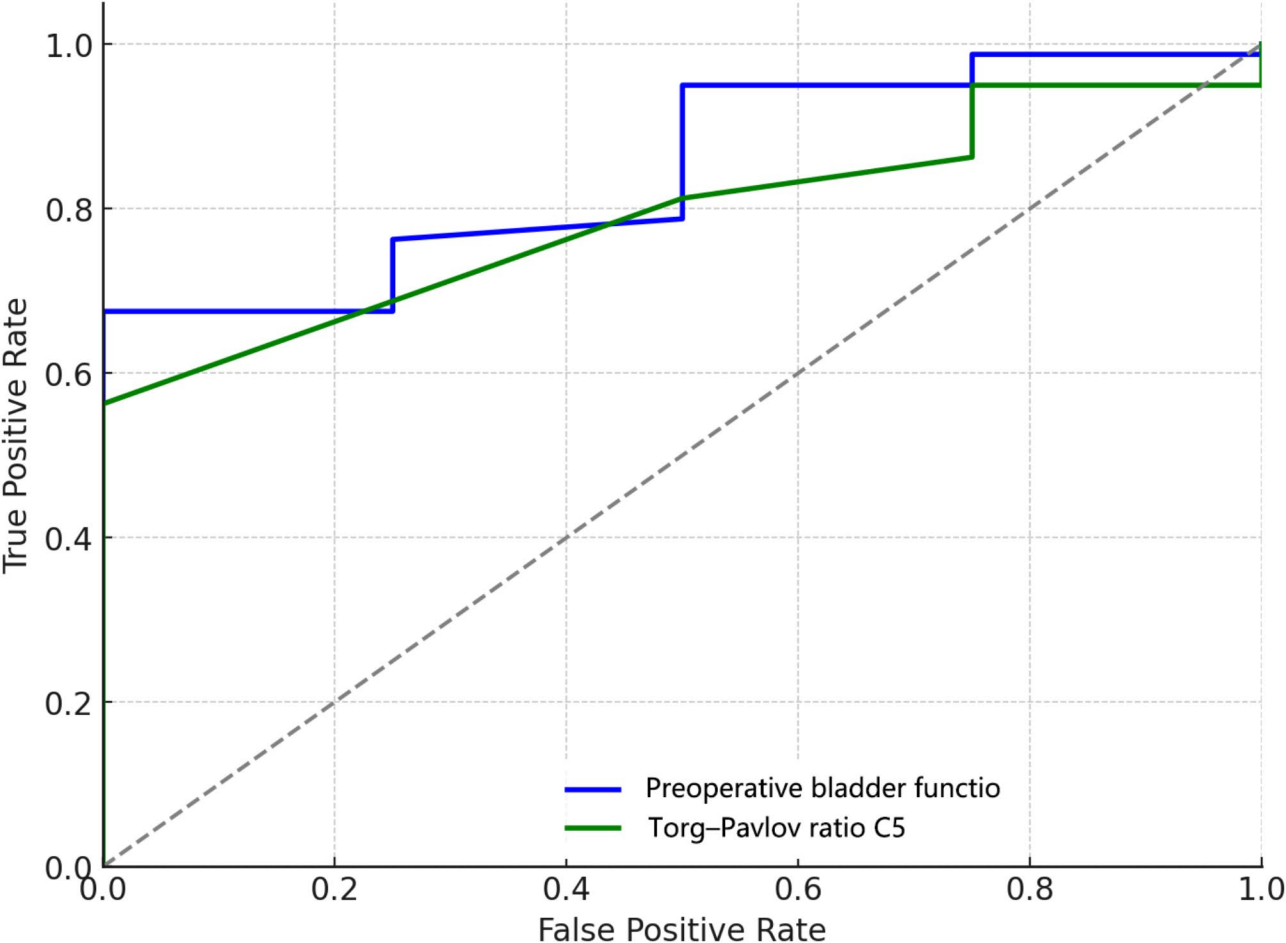
ROC curves for bladder function recovery after LAMP surgery

Table 6; Fig. 5 reveal factors influencing bladder function recovery. Preoperative bladder status emerged as a key predictor (OR = 1.51, AUC = 0.85), with higher scores indicating better outcomes. Similarly, the Torg–Pavlov ratio at C5 (OR = 0.36, AUC = 0.79) suggested that narrower spinal canals are associated with improved recovery, although bladder function alterations were generally less pronounced in the study population.

**Table 6:** Binary logistic regression for factors influencing bladder function after LAMP surgery

Variable	Odds Ratio	Coefficient	AUC
Preoperative bladder function	1.51	0.41	0.85
Torg–Pavlov ratio C5	0.36	-1.02	0.79

AUC area under the curve

#### QOL

**Fig. 6:**
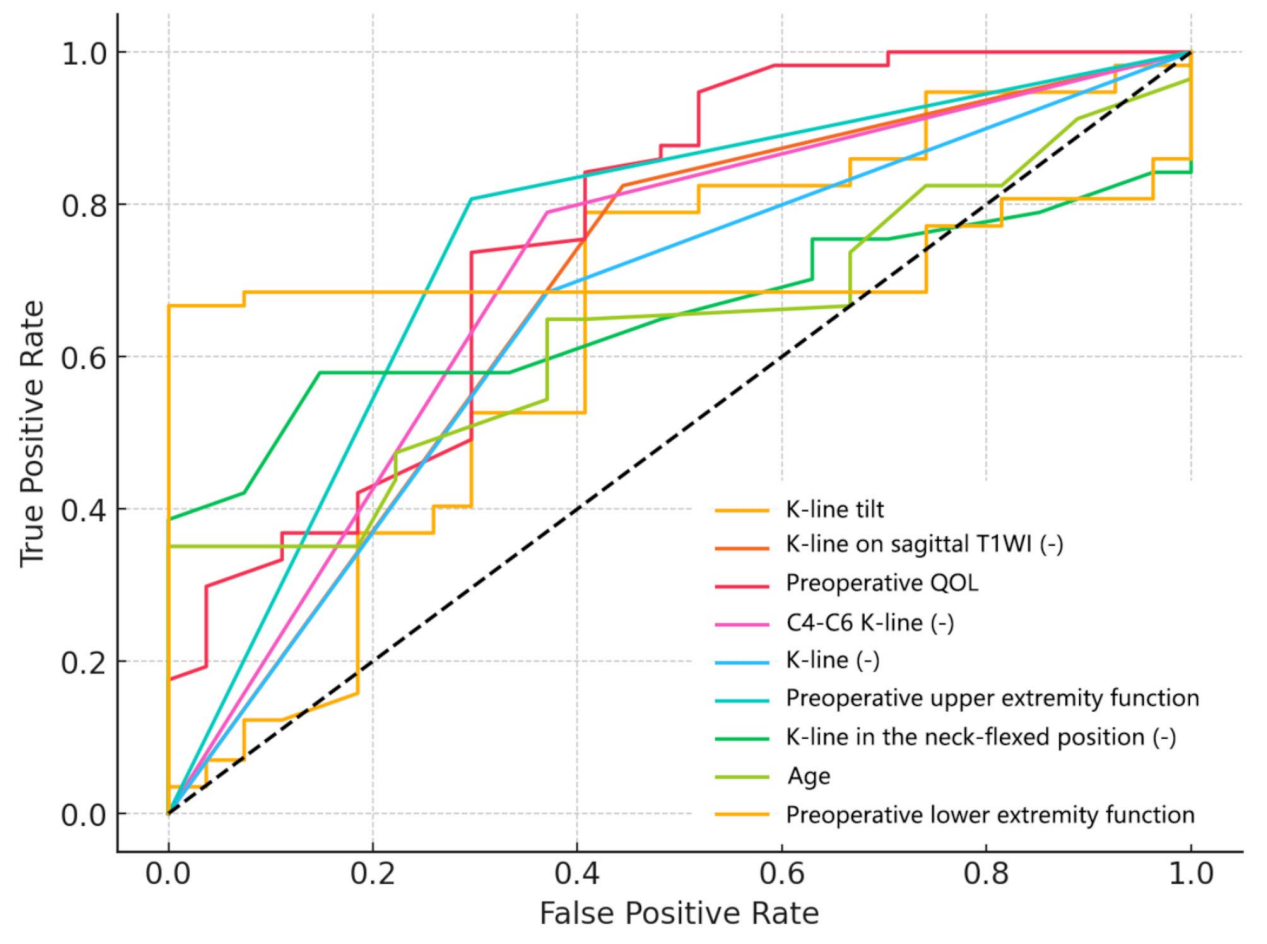
ROC curves for qol recovery after LAMP surgery. QOL quality of life; T1WI T1-weighted imaging

Table 7; Fig. 6 present the results of a binary logistic regression analysis and ROC curves assessing factors influencing QOL recovery after LAMP surgery. K-line on sagittal T1WI and K-line tilt showed the strongest predictive value, with a negative coefficient, indicating that negative findings correlate with poorer QOL recovery. Similarly, C4-C6 K-line (OR 0.23, AUC 0.71) was associated with worse outcomes. Preoperative QOL emerged as a strong positive predictor, with an OR of 2.68 and a coefficient of 0.99, underscoring the association between better preoperative QOL and improved recovery.

**Table 7:** Binary logistic regression for factors influencing QOL function after LAMP surgery

Variable	Odds Ratio	Coefficient	AUC
K-line tilt	0.31	-1.16	0.76
K-line on sagittal T1WI (-)	0.17	-1.80	0.76
Preoperative QOL	2.68	0.99	0.71
C4-C6 K-line (-)	0.23	-1.47	0.71
K-line (-)	0.25	-1.40	0.69
Preoperative upper extremity function	1.86	0.62	0.66
K-line in the neck-flexed position (-)	0.35	-1.06	0.66
Age	0.61	-0.49	0.64
Preoperative lower extremity function	1.72	0.54	0.64

QOL quality of life; T1WI T1-weighted imaging; AUC area under the curve

## Discussion

This study evaluated the predictive value of various preoperative factors, including K-line measurements, SCOR, the Torg-Pavlov ratio, shape of COPLL, complications and JOACEMQ domain scores, on recovery outcomes in patients with COPLL who underwent LAMP. Our findings indicate that certain K-line parameters, particularly the K-line-related parameters are closely associated with improvements in cervical spine function, upper extremity function, lower extremity function, as well as QOL. Notably, JOACMEQ domain scores also exhibited robust predictive capability, particularly for upper and lower extremity function, bladder function and QOL. The Torg-Pavlov ratio and shape of COPLL showed moderate predictive value for certain outcomes, such as upper and lower extremity function. Overall, these results highlight the varying degrees of influence that different preoperative factors, including functional assessments and anatomical parameters, have on surgical outcomes. By identifying which factors most strongly predict specific recovery domains, this study provides valuable insights into optimizing patient selection and tailoring surgical planning for COPLL.

### K-line-related parameters and surgical outcomes

Our study demonstrates that K-line-related parameters are significantly associated with recovery in cervical spine function, spinal cord and neurological function, as well as QOL in patients with COPLL undergoing LAMP. These findings are consistent with previous research, which has shown that K-line positive COPLL patients typically experience higher JOA scores, lower NDI scores, and improved SF-36 PCS scores following LAMP surgery [15–18]. In recent years, there have been several reports identifying K-line tilt as an important predictor. Wei et al. reported that K-line tilt was significantly correlated with postoperative JOA scores and had the greatest influence among sagittal parameters such as C2–C7 SVA and T1 slope [19].

Other modified K-line parameters, such as C4-C6 K-line, K-line in the neck-flexed position, and K-line on sagittal T1WI, have been predominantly studied in K-line (−) patient subgroups [3, 20, 21]. These subgroup analyses focus on patients with severe spinal cord compression but often neglect the distribution and predictive reliability of these modified parameters in K-line (+) patients. Whether these modifications may inadvertently increase false-positive rates, thereby reducing the overall predictive capability for surgical outcomes, remains unclear.

To our knowledge, this study is the first to directly compare the predictive value of K-line and its modified parameters for surgical outcomes in COPLL patients undergoing LAMP. This comprehensive approach provides new insights into the differential roles of these parameters in optimizing surgical planning and patient selection.

### Preoperative scores and surgical outcomes

Previous studies on the relationship between preoperative scores and functional outcomes have been inconclusive. However, consistent with our findings, most studies indicate a positive correlation between higher preoperative scores and better postoperative recovery. For instance, Gu et al. demonstrated that higher preoperative JOA scores significantly predicted favorable outcomes [22], and Nakashima et al. emphasized their importance in recovery rates for cervical OPLL [23]. In contrast, Hartman et al. [24]. reported greater absolute improvement in patients with severe preoperative dysfunction (NDI ≥ 50) compared to those with mild-to-moderate dysfunction (NDI < 50) following cervical disc replacement, likely due to the statistical “ceiling effect,” where less dysfunction leaves less room for measurable improvement [25]. Our criteria, which include achieving a postoperative score exceeding 90, mitigate this effect and provide a more accurate assessment of surgical efficacy.

However, we found that the predictive value of preoperative scores varies across different domains. For instance, domains assessing cervical spine function, upper and lower extremity motor function, and QOL showed strong correlations with postoperative improvement. In contrast, scores related to bladder function exhibited a weaker relationship with surgical outcomes. This difference may be attributed to the multifactorial nature of bladder dysfunction or to the relatively infrequent occurrence of bladder dysfunction in COPLL patients, which may result in insufficient statistical power.

### SCOR and Pavlov ratio as predictors of surgical outcomes

Our study shows that SCOR and Torg-Pavlov ratio are useful imaging parameters for predicting surgical outcomes in COPLL patients undergoing LAMP, with higher SCOR and lower Torg-Pavlov ratio linked to poorer recovery. These findings align with previous research. For instance, Nakashima et al. reported that in patients undergoing LAMP, a SCOR greater than 60% significantly increased the risk of poor neurological recovery [23]. Similarly, Wang et al. identified a Pavlov ratio < 0.65 as a significant risk factor for postoperative C5 palsy, highlighting its importance in assessing surgical risks and outcomes [26].

Despite their value, our findings indicate that SCOR and Pavlov ratio are not primary predictors of recovery across all functional domains. Instead, their utility may lie in providing supplementary information about spinal canal anatomy and the feasibility of certain surgical approaches. This observation aligns with the work of Nouri et al., who noted that while SCOR ≥ 70% effectively identified patients with congenital cervical stenosis and correlated with baseline neurological deficits, its influence on long-term surgical outcomes was less significant compared to other factors [27].

These results suggest that SCOR and Pavlov ratio should be integrated with other predictive tools, such as K-line parameters and preoperative functional scores, to develop a comprehensive framework for guiding surgical decisions and managing patient expectations.

### Shape of COPLL and surgical outcomes

Our study suggests that a hill-shaped COPLL is associated with poorer recovery of both upper and lower extremity function following LAMP surgery. This finding is supported by existing literature. Nakashima et al. [28] reported that patients with hill-shaped OPLL often experience more severe spinal cord compression, which negatively impacts postoperative neurological recovery. They proposed that this OPLL morphology is typically linked to a higher canal-occupying ratio and greater compression, which restricts blood flow to the spinal cord and nerve roots, thereby hindering recovery and functional outcomes. Similarly, Lindsay et al. [29] identified hill-shaped OPLL as a complex variant with a higher canal-occupying ratio, which complicates surgical decompression and ultimately impedes postoperative recovery.

### Advantages of using JOACMEQ as an evaluation tool and outcome measure

We selected the JOACMEQ as the primary evaluation tool and outcome measure in this study due to its comprehensive, patient-centered design and strong psychometric validation. Unlike the traditional JOA score, which primarily evaluates neurological function, JOACMEQ assesses five domains: cervical spine function, upper extremity function, lower extremity function, bladder function, and QOL [30]. This multidimensional framework provides a more holistic evaluation of patient recovery, incorporating self-reported outcomes that reduce observer bias and enhance sensitivity to subtle functional improvements, particularly in QOL and upper extremity function [31, 32]. Studies have shown strong correlations between JOACMEQ and other validated tools, such as the SF-36 and NDI, further confirming its reliability and validity [33]. Additionally, its concise format simplifies patient evaluation, reduces follow-up loss, and facilitates streamlined data collection, making it highly suitable for clinical and research purposes.

### Limitations

This study has several limitations. First, its retrospective design and reliance on a cohort from four medical centers may introduce selection bias and limit the generalizability of the findings. Second, the sample size, while adequate, may not fully capture variability in outcomes, particularly for less common parameters such as bladder function recovery. Third, the follow-up period, although sufficient to assess immediate postoperative outcomes, may not reflect long-term recovery trajectories. Finally, while the JOACMEQ offers a multidimensional assessment, its reliance on patient-reported data may introduce response bias, particularly in QOL measures. Future studies with larger, multicenter prospective cohorts and longer follow-up durations are warranted to validate these findings.

## Conclusion

Preoperative parameters play a crucial role in predicting postoperative outcomes following LAMP surgery for myelopathy caused by COPLL. Key predictors, including K-line-related parameters and preoperative functional scores, significantly influence recovery across various functional domains, and other factors like K-line tilt, hill-shaped COPLL and maximum SCOR also provide valuable insight into recovery potential. These findings emphasize the importance of assessing preoperative factors for tailored surgical planning and improved postoperative expectations, particularly for individuals with varying degrees of baseline functional status.

## Data Availability

The datasets used and analyzed during the current study are available from the corresponding author on reasonable request.
